# A Meta-Analysis of Short-Term Outcomes of TAVR versus SAVR in Bicuspid Aortic Valve Stenosis and TAVR Results in Different Bicuspid Valve Anatomies

**DOI:** 10.3390/jcm12237371

**Published:** 2023-11-28

**Authors:** Riccardo Improta, Gianluca Di Pietro, Novis Kola, Lucia Ilaria Birtolo, Riccardo Colantonio, Emanuele Bruno, Marco Tocci, Alessandra Giansante, Michele Sannino, Veronica Zullino, Sara Monosilio, Sara Cimino, Viviana Maestrini, Paolo Severino, Roberto Badagliacca, Carlo Lavalle, Paola Celli, Wael Saade, Carmine Musto, Fabrizio D’Ascenzo, Fabio Miraldi, Carmine Dario Vizza, Gennaro Sardella, Massimo Mancone

**Affiliations:** 1 Department of Clinical, Internal, Anesthesiology and Cardiovascular Sciences, Umberto I Hospital, Sapienza University of Rome, 00161 Roma, Italy; riccardo.improta@uniroma1.it (R.I.); gianluca.dipietro@uniroma1.it (G.D.P.); kola.1784784@studenti.uniroma1.it (N.K.); luciailaria.birtolo@uniroma1.it (L.I.B.); marcotocci1392@gmail.com (M.T.); alessadra.giansante@uniroma1.it (A.G.); michele.sannino@uniroma1.it (M.S.); sara.monosilio@uniroma1.it (S.M.); sara.cimino@uniroma1.it (S.C.); viviana.maestrini@uniroma1.it (V.M.); paolo.severino@uniroma1.it (P.S.); roberto.badagliacca@uniroma1.it (R.B.); carlo.lavalle@uniroma1.it (C.L.); wael.saade@uniroma1.it (W.S.); fabio.miraldi@uniroma1.it (F.M.); dario.vizza@uniroma1.it (C.D.V.); rino.sardella@uniroma1.it (G.S.); 2 Anesthesia and Resuscitation in Specialistic Surgeries and Transplants, Umberto I Hospital, 00161 Roma, Italy; v.zullino@policlinicoumberto1.it (V.Z.); paola.celli@uniroma1.it (P.C.); 3 San Camillo-Forlanini Hospital, 00152 Rome, Italy; c.musto@hotmail.it; 4 Department of Medical Science, Division of Cardiology, Molinette Hospital, Turin University, 10126 Torino, Italy; fabrizio.dascenzo@gmail.com

**Keywords:** bicuspid aortic valve, TAVR, SAVR, type, outcomes

## Abstract

Background: To provide a comprehensive analysis of the current literature comparing the outcomes of surgical aortic valve replacement (SAVR) and transcatheter aortic valve replacement (TAVR) in patients with bicuspid aortic stenosis (BAS), with particular attention to BAV morphology in patients undergoing TAVR. Methods: Following PRISMA guidelines, all relevant articles with no design restrictions from PubMed, CCTR (Cochrane Controlled Trials Register), and Google Scholar were screened for inclusion. Studies were included if they reported clinical endpoints for SAVR and TAVR or, in BAS treated with TAVR, for type 1 and non-type 1 morphology. Odds ratio and Cohen’s D were considered as effect size measurements for qualitative and quantitative variables, respectively. Results: A total of eight studies comparing short-term outcomes between SAVR and TAVR and nine studies with outcomes data between type 1 and non-type 1 BAS treated with TAVR were considered for the final analysis. No statistically significant difference was found for what concerns the rates of death, stroke, and acute kidney injury between SAVR and TAVR. In comparison to patients undergoing SAVR, the incidence of PPI (permanent pacemaker implantation) was greater in the TAVR group (OR 0.35, 95% CI 0.15–0.79, *p* = 0.01), and the frequency of bleeding events was found to be higher among patients undergoing SAVR (OR 4.3, 95% CI 2.9–6.4, *p* < 0.001). The probabilities of 30-day mortality, stroke, and any bleeding were not significantly affected by bicuspid valve morphology in TAVR patients. PPI or development of new conduction anomalies was found to be more frequent in type 1 anatomies (OR 0.46, 95% CI 0.30–0.70, *p* <0.001). Mildly lower post-procedural transprothesic gradients were found in patients with type 1 morphology. Conclusions: In BAS patients, TAVR has comparable short-term outcomes rates with SAVR, but higher PPI rates and lower incidence of bleeding events. In patients undergoing TAVR, type 1 BAS is associated with lower postoperative transvalvular gradients but higher PPI rates and conduction abnormalities

## 1. Introduction

A bicuspid aortic valve (BAV) is the most common congenital abnormality, occurring in 1–2% of the population [1]. While the precise etiology is not fully understood, studies suggest a strong genetic component, with multiple genes implicated in valve formation and function [2]. Sievers’ classification system is based on the raphes’ cusp size, number, and position [3]. Type 1 (one raphe) with right–left (R-L) coronary cusps fusion is the most common, with 70–80% prevalence [4]. The clinical presentation of bicuspid aortic stenosis has an earlier onset than that of patients with a tricuspid aortic valve (TAV) [5]. Indeed, increased shear stress through the valve caused by higher leaflet coaptation points and asymmetrical BAV leaflet motion can lead to calcification at a young age [6]. Surgical aortic valve replacement (SAVR) remains the default strategy in this selected cohort of patients with low in-hospital mortality rates and excellent long-term outcomes [7,8,9,10,11,12]. Transcatheter aortic valve replacement (TAVR) has been emerging as the first-line treatment in patients with symptomatic severe tricuspid aortic valve (TAV) stenosis across all surgical risk categories [13,14,15,16,17,18]. However, it is not yet recommended for BAV stenosis patients [7], even if some observational studies have reported its safety and feasibility. Bicuspid valve degeneration and the surrounding structures might be significantly different from a tricuspid valve: aortic structures, depending on valve morphology, are generally larger in BAV and, along with anomalies in supra-annular geometry, are a concern in valve sizing and deployment. Moreover, stenotic leaflets present higher and asymmetrically distributed calcium, and there may be coronary-origin anomalies or ostia located closer to the commissures increasing the risk of coronary obstruction. For the aforementioned factors, we hypothesized that valve anatomy could have an impact on procedural and short-term outcomes. Because of the extending indication of TAVR to a population with lower surgical risk profiles and younger patients, operators should evaluate all the procedural characteristics, aiming to obtain the best result even in such complex scenarios. As previously stated, Sievers type 1 is the most common in the general population, so a comparison with the other two groups could highlight differences in procedural outcomes.

This systematic meta-analysis aimed to compare the short-term outcomes of patients treated with TAVR versus SAVR in BAV patients and, between patients undergoing TAVR, short-term outcomes of type 1 and non-type 1 BAV morphology.

## 2. Materials and Methods

### 2.1. Eligibility Criteria, Databases, and Search Strategy

The analysis was performed following the preferred reporting items for systematic reviews and meta-analyses (PRISMA) [19] guidelines, and the study was registered on PROSPERO (CRD42023430833). The following online databases were evaluated for articles published by 30 October 2022: PubMed/MEDLINE, CENTRAL/CCTR (Cochrane Controlled Trials Register), Google Scholar, and references from relevant articles. The subsequent terms, following a PICO strategy (population, intervention, control, and outcomes), were searched in different combinations: “BAV,” “bicuspid aortic valve”, “transcatheter aortic valve replacement”, “TAVR”, “transcatheter aortic valve implantation”, “TAVI”, “surgical aortic valve replacement”, “SAVR”, “bicuspid anatomy”, “bicuspid type”, “bicuspid morphology”, “outcomes”.

For the final inclusion in the analysis, titles of records were identified through the database search, followed by the removal of duplicates. Abstracts were selected and, after analysis of full texts, when available, screened for eligibility.

**Figure 1:**
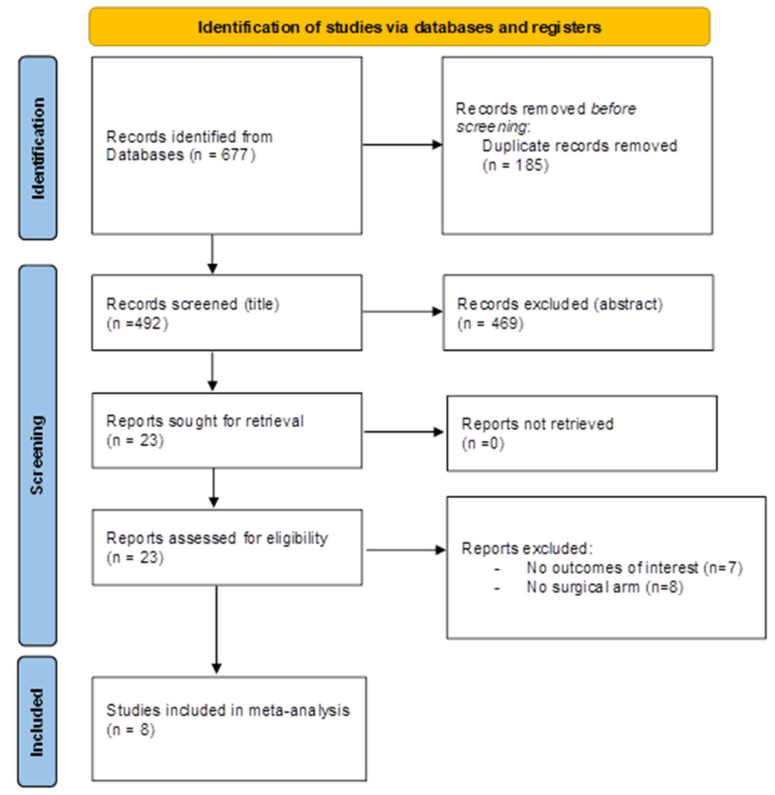
Flow diagram for research of relevant articles for outcomes between TAVR and SAVR in BAV. Transcatheter aortic valve replacement = TAVR, surgical aortic valve replacement = SAVR, bicuspid aortic valve = BAV.

**Figure 2:**
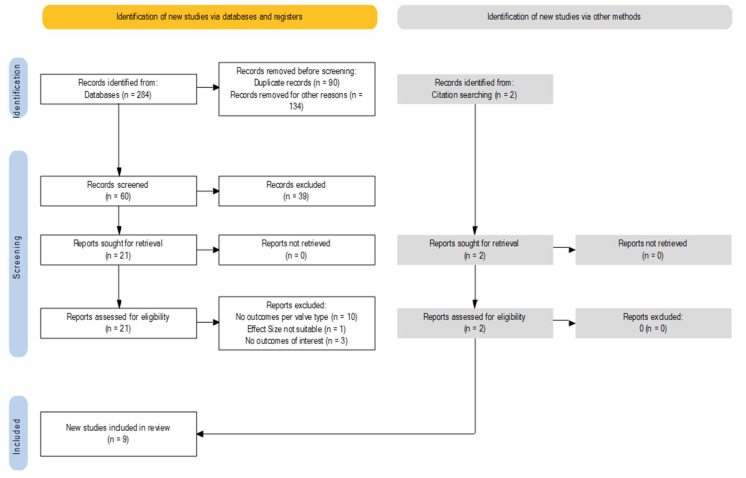
Flow diagram for research of relevant articles for outcomes between type 1 and non-type 1 in BAV undergoing TAVR. Transcatheter aortic valve replacement = TAVR, bicuspid aortic valve = BAV.

Studies were included if the following inclusion criteria were fulfilled: (1) the population included BAV patients with severe AS; (2) one group underwent TAVI, and a second one went through SAVR or if, in a TAVR cohort, a distinction was made regarding types of bicuspid valve morphology; (3) the evaluated outcomes included in-hospital or 30-day follow-up death, stroke, acute kidney injury (AKI), bleeding events, new permanent pacemaker implantation (PPI), length of hospital stay, peri-valvular leakage (PVL), aortic injury, need for ViV (valve-in-valve) or cardio surgery intervention, mean trans-prothesis gradient, and coronary obstruction. The study design was deemed to be irrelevant. A flow diagram is reported in Figure 1 and Figure 2. Ethical approval was not requested, and no language restriction was applied.

### 2.2. Assessment of Risk of Bias

The admitted studies underwent stratification for risk of bias through the risk of bias in nonrandomized studies of interventions tool (ROBINS-I) [20]. A severity scale was used to identify in each domain and in the overall analysis the low, moderate, and serious risks of bias; in the end, the studies and their characteristics were classified into mild, moderate, and serious risk of bias. Two independent reviewers assessed the risk for bias. When there was a disagreement, a third reviewer made the final decision.

### 2.3. Statistical Analysis

Data inference was undertaken only if three studies for each outcome were found to have at least one event in a sub-group.

Statistical heterogeneity was assessed through the inconsistency index I^2^. A test for heterogeneity was then performed: whenever the alternative hypothesis of heterogeneity was satisfied (*p* < 0.05), a random-effect model was used to extrapolate data from the chosen studies; elsewhere, a fixed-effect model was adopted.

To assess the publication bias, a funnel plot was generated for each outcome, and it was statistically assessed by the Egger’s test if feasible: whenever the alternative hypothesis was accepted, each study was re-evaluated and, if deemed to significantly determine publication bias, removed from the analysis until Egger’s test demonstrated absence of bias. Publication bias for binary outcomes was also evaluated through the Harbord and Peters test and only if the null hypothesis was accepted in two out of three tests (*p* > 0.05) was the analysis deemed to be free from publication bias.

Odds ratios with 95% confidence intervals (CIs) were calculated for the effect size for short-term binary outcomes, and forest plots were used to represent differences in clinical endpoints. For quantitative data effect size, Cohen’s D with 95% CIs and *p*-values were considered. A significant cut-off value of less than 0.05 was chosen to identify statistical relevance. All analyses were performed with SPSS (version 29.0, IBM).

## 3. Results

### 3.1. Study Selection and Characteristics

For short-term outcomes between TAVR and SAVR patients, after the aforementioned described search and selection criteria, eight studies [21,22,23,24,25,26,27,28] were considered eligible for the analysis (Table 1).

**Figure 3:**
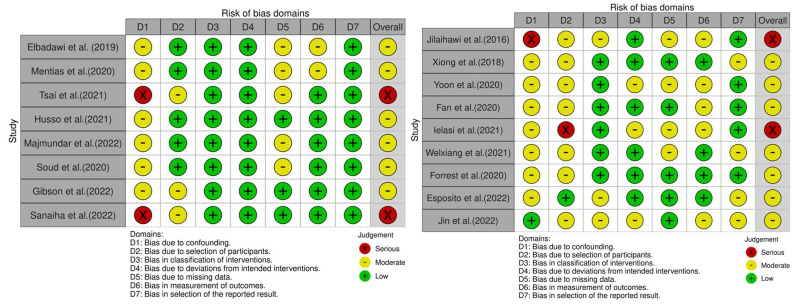
Qualitative assessment for bias. From left to right, respectively: analysis of bias for different domains for TAVR vs. SAVR and TAVR in different anatomies; studies listed in Table 1.

The totality of the data comes from nonrandomized observational retrospective studies. Specifically, five of them had propensity score-matched populations, and one had populations matched for age and sex only; five studies were conducted in the United States. The analyzed population consisted of 62,981 patients, of whom 7152 underwent TAVR and 55,829 were treated with SAVR. After searching for studies evaluating short-term outcomes between different types of bicuspid anatomies undergoing TAVR, nine studies [18,29,30,31,32,33,34,35,36] were included in the final analysis (Table 1). The totality of the studies was nonrandomized, retrospective, and observational in design, and none had the propensity score matching performed. Finally, the total analyzed population included 2099 patients, of which 470 had a type 0 or type 2 morphology and 1629 a type 1 anatomy. Patients’ characteristics from each study, exclusion criteria, and outcomes definitions used in the selected studies are listed in the Supplementary material (Tables S1 and S2). Qualitative assessment for bias of the studies with the ROBINS-I tool is shown in Figure 3. Even when propensity score matching was performed, we cannot exclude that some confounders may have not been considered, so every study has at least a moderate risk for confounding bias; moreover, often, pre-operative risk scores were not available, and the risk for selection bias could be seriously high in different studies.

### 3.2. Analysis of Short-Term Outcomes

**Figure 4:**
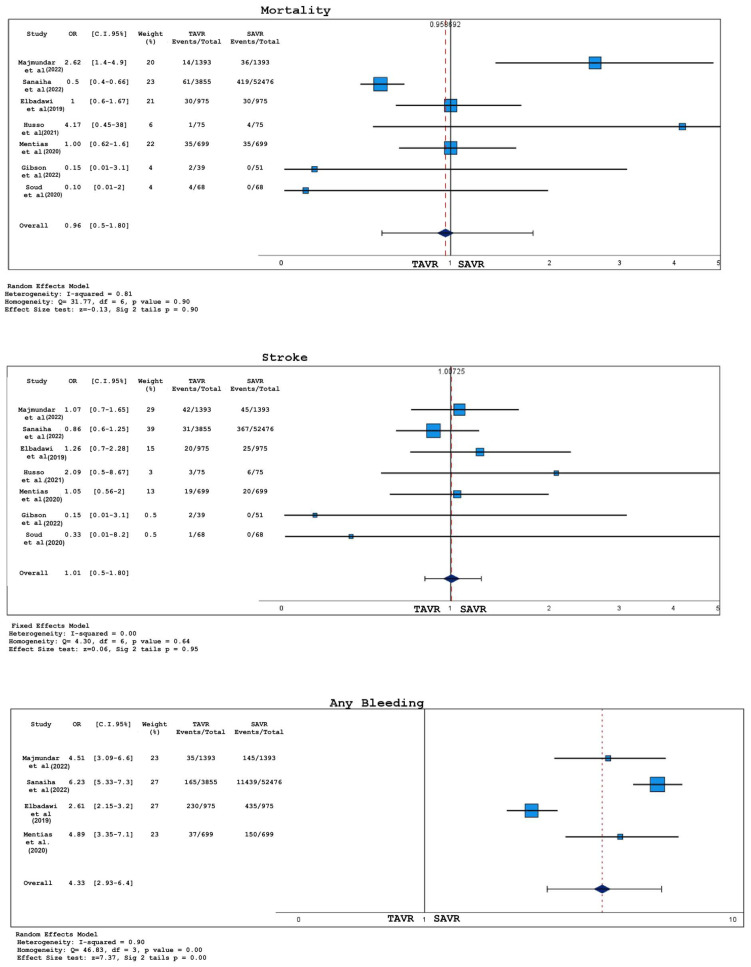
Forest plots for 30-day mortality, stroke, and any bleeding for TAVR vs. SAVR; studies listed in Table 1.

**Figure 5:**
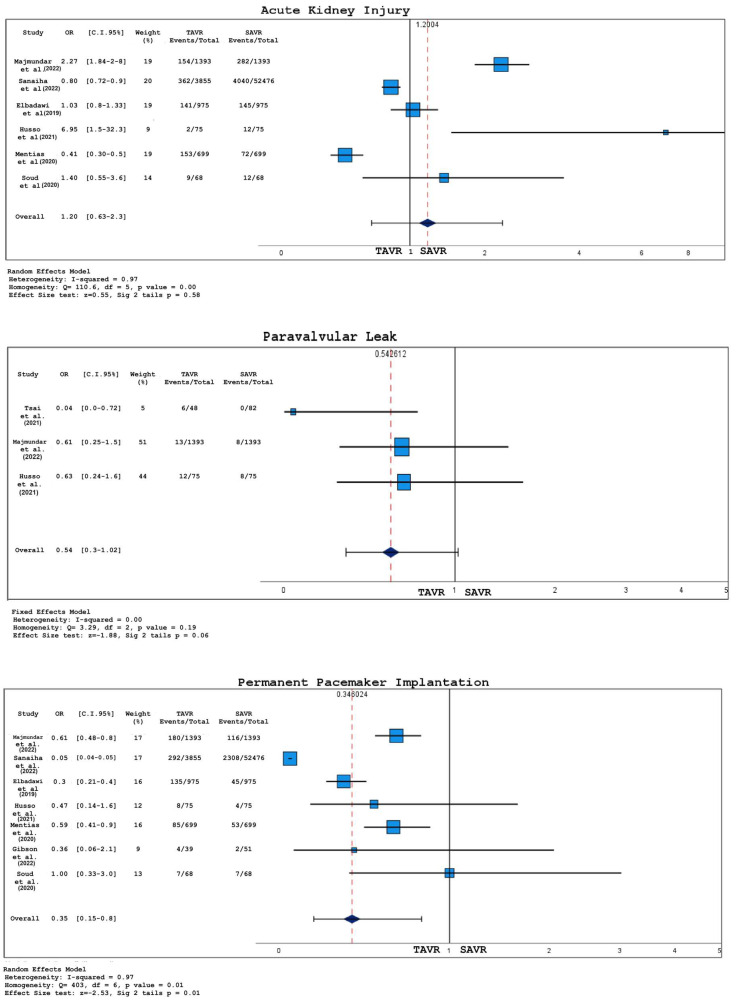
Forest plots for 30-day acute kidney injury, paravalvular leak, and permanent pacemaker implantation for TAVR vs. SAVR; studies listed in Table 1.

Forest plots for short-term outcomes (including both in-hospital and 30-day events) between TAVR and SAVR in bicuspid aortic valve patients are represented in Figure 4 and Figure 5.

No statistically significant difference was found for what concerns the rates of death (OR 0.96, 95% CI 0.51–1.80, *p* = 0.90), stroke (OR 1.01, 95% CI 0.8–1.27, *p* = 0.95), and acute kidney injury (OR 1.2, 95% CI 0.63–2.30 *p* = 0.581).

In comparison to patients undergoing SAVR, the incidence of PPI was greater in the TAVR group (OR 0.35, 95% CI 0.15–0.79, *p* = 0.01).

After the assessment of publication bias through funnel plots and Egger’s test, four out of eight studies were excluded from the analysis for any bleeding events; the exclusion process was carried out as aforementioned. Finally, the frequency of any bleeding event was higher among patients undergoing SAVR (OR 4.3, 95% CI 2.9–6.4, *p* < 0.001).

Post-operative occurrence of peri-valvular leakage of any degree was reported in three papers out of eight: no statistically significant difference was observed between the two treatments (OR 0.5, 95% CI 0.29–1.02, *p* = 0.06) even if a trend toward a higher incidence of PVL in TAVR group was observed.

Length of stay was not significantly affected by the treatment group, but a trend close to a shorter hospitalization was found in TAVR patients (Cohen’s D −0.72, 95% CI −1.45/0.00, *p* = 0.051).

**Figure 6:**
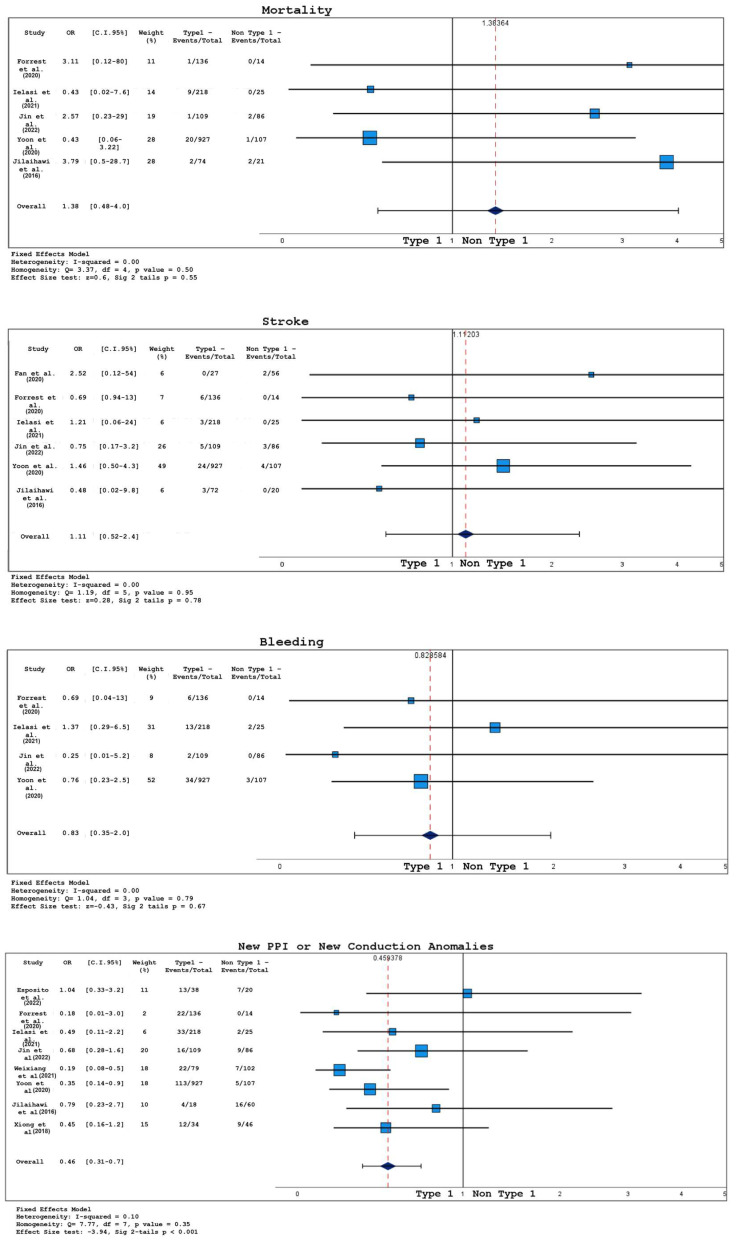
Forest plots for 30-day outcomes in type 1 BAV vs. non-type 1 BAV. PPI = permanent pacemaker implantation; studies listed in Table 1.

**Figure 7:**
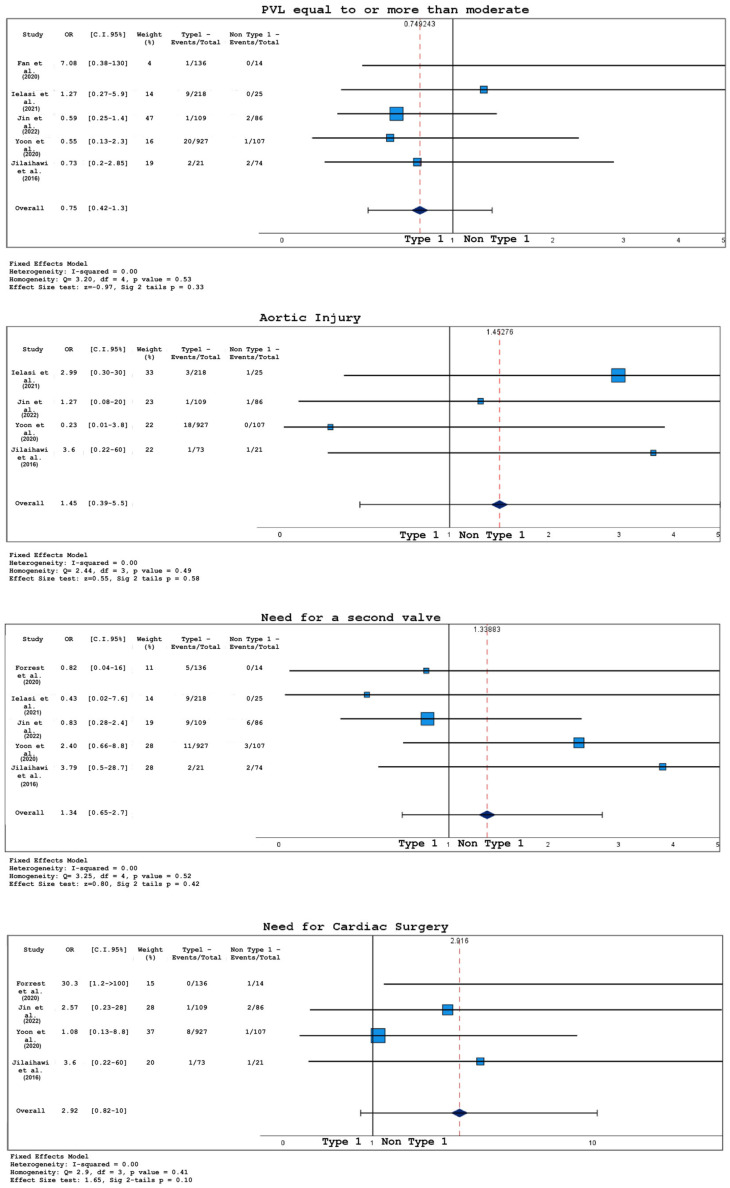
Forest plots for 30-day outcomes in type 1 BAV vs. non-type 1 BAV. PVL = paravalvular leak; studies listed in Table 1.

Pre-operative risk scores (STS PROM or EUROSCORE II) were available only in two out of eight studies, so it was not possible to extrapolate any conclusive data. Analysis of short-term outcomes in bicuspid patients undergoing TAVR concerning valve anatomy (comparing type 1 and non-type 1 morphology) are plotted in Figure 6 and Figure 7.

The probabilities of 30-day mortality (OR 1.38, 95% CI 0.48–4.03, *p* = 0.55), stroke (OR 1.11, 95% CI 0.52–2.36, *p* = 0.78), or any bleeding (OR 0.83, 95% CI 0.35–1.97, *p* = 0.67) were not significantly affected by valve morphology.

While moderate–severe peri-valvular leakage incidence was not different between the two groups (OR 0.75, 95% CI 0.42–1.34, *p* = 0.33), permanent pacemaker implantation or development of new conduction anomalies were found to be more frequent in type 1 anatomies (OR 0.46, 95% CI 0.30–0.70, *p* <0.001). Mildly lower post-procedural transprothesic gradients were found in patients with type 1 morphology (Cohen’s D −0.19, 95% CI −0.34/−0.04, *p* = 0.01).

For what concerns peri-procedural complications, the probabilities of needing a second valve (OR 1.34, 95% CI 0.65–2.7, *p* = 0.42), conversion to cardiac surgery (OR 2.92, 95% CI 0.82–10.0, *p* = 0.10), and aortic root injury (OR 1.45, 95% CI 0.39–5.5, *p* = 0.58) were not significantly influenced by BAV structure.

Data for coronary artery obstruction were not sufficient to draw unbiased and precise conclusions: in three out of four analyzed studies, no events were recorded in the two groups.

## 4. Discussion

The main findings of our meta-analysis can be summarized as follows:

1. There was no significant difference in 30-day deaths, stroke, or acute kidney injury (AKI) between TAVR and SAVR;
2. Bleedings were more common in the SAVR group;
3. Patients with BAS who underwent TAVR had a higher probability of permanent pacemaker implantation (PPI) compared to those treated with SAVR;
4. Even when not reaching statistically significant thresholds, TAVR was associated with a major incidence of paravalvular leakage and a shorter hospital stay, as opposed to SAVR;
5. According to the Sievers classification, TAVR patients with type 1 BAV had lower post-operative transvalvular gradients but an increased risk of PPI and new conduction abnormalities compared to those with type 0 or type 2.

Bicuspid aortic stenosis (BAS) is a challenge for interventional cardiologists due to its unfavorable morphological features [37]. The often more elliptical annulus and calcified raphes may predispose to an increased risk of periprocedural complications or failure. These challenges explain the exclusion of BAV patients from the larger pivot clinical trials and the strong recommendation of international guidelines for surgery as first-line therapy [7,38]. Despite the upcoming and promising results of TAVR in lower-surgical-risk patients or younger patients with tricuspid aortic stenosis [15,17], the currently available evidence about TAVR in BAV patients is weak and is based mainly on observational [23,26,29,39,40,41] or registry studies [42,43,44] with limited data about outcomes. For this reason, Nuyens et al. presented a study design proposal for a dedicated randomized controlled trial (RCT) about TAVR versus SAVR in bicuspid patients [45].

Consistent with previous studies [23,26,46,47,48,49], our analysis demonstrated that performing TAVR in BAS patients resulted in comparable rates of 30-day all causes of death, stroke, and AKI with SAVR supporting the short-term feasibility of the percutaneous approach in this selected cohort of patients. Our findings complement previous data from other studies comparing TAVR outcomes in bicuspid and tricuspid aortic stenosis, providing additional reassuring information about TAVR in BAS [50,51,52].

More specifically, stroke is a common complication for both TAVR and SAVR. Because BAVs have a higher calcium burden, frequent pre-dilation maneuvers during transcatheter implantation may explain the reported 30-day stroke rates [53]. Similarly, the cross-clamping aorta during SAVR favors the dislodgement of loose atheromatous plaque or mural emboli [54].

Previous studies on both BAV and non-BAV patients have shown that the invasiveness of the surgical approach led to a higher incidence of bleeding [26,55]. This aligns with the findings of our current study.

Younger patients undergoing TAVR are at risk of increased mortality and re-hospitalizations due to significant concerns with PPI and PVLs [56,57]. A shorter membranous septum and the proximity of the raphe to the atrioventricular node can predispose patients with BAV to PPI [31,58]. Conversely, PVLs are correlated with asymmetric cusp calcification, device landing zone calcification, and an oval-shaped annulus [59].

Therefore, selecting the appropriate valve type and size can minimize adverse events. Balloon-expandable valves with higher radial force can provide optimal sealing and reduce PVLs. Aggressive oversizing should be avoided to reduce interaction with the conductance system and the need for post-operative PPI. Nonetheless, it has been proposed that an inverse correlation exists between oversizing and PVLs, necessitating a careful balance of the risks of PPI and PVLs in BAV morphology [60].

Our analysis has conclusively shown that managing TAVR patients requires careful consideration of the length of stay (LOS). It is a critical factor that influences both healthcare costs and patient outcomes [61]. Our analysis demonstrated a trend through a shorter LOS for patients with BAS who underwent TAVR compared to SAVR, as previously reported [48]. Conscious sedation, limited circulatory support, and smaller sheath sizes impact the LOS of TAVR patients [61].

Bicuspid aortic valve anatomy presents some complex aspects that must be considered during the pre-procedural planning of TAVR, and that can impact the technical success and short-term outcomes.

Type 1 anatomy was the most represented in our analysis in agreement with epidemiological data [62], and for what concerns the subtypes of cusps fusion, type 1 L-R was the most frequent (244 patients out of 303 reported cases: about 80%). Permanent pacemaker implantation was strongly related to type 1 anatomy: the counterblow exerted through the prothesis frame by the raphe calcification between the right and left coronary cusp on the non-coronary cusp has been advocated to be a rationale for the compression of the His bundle and subsequent development of conduction anomalies and pacemaker implantation [53]. Even in previous TAVR in tricuspid aortic valve studies, left coronary cusp calcification was associated with higher rates of pacemaker implantation, supporting this mechanism for the development of conduction anomalies [63,64].

Type 0 BAV represents a challenging scenario for prothesis sizing, as already explored by Frangieh et al. [65], because of the lack of the three hinge points usually needed for accurate valve dimensioning. This could lead to prothesis under-sizing and consequent high post-procedural trans-prothesis gradients, as observed in our analysis and also by Bugani et al. [66], who reported device failure with post-procedural high residual gradient (mean gradient ≥ 20 mmHg) in type 0 BAV after prothesis implantation. This finding did not impact the risk for the more than moderate paravalvular leakage that appeared to be similar between the two groups.

A previous meta-analysis by Du et al. [67] showed similar results for what concerns the 30-day clinical endpoint between BAV subtypes such as mortality, stroke, and life-threatening bleeding, even if fewer studies were considered in the overall analysis. Thirty-day stroke was found to be significantly higher in BAV patients undergoing TAVR with respect to patients with TAV [68]: the larger extent of calcifications and the need for pre- and post-dilation in BAV anatomies are advocated as being responsible for this finding. In a recent study by Zhang et al. [69] in BAV patients, the use of cerebral embolic protection devices (CEPDs) led to a significantly lower incidence of procedural stroke, whereas in TAV patients the evidence about the utility of CEPDs is conflicting: BAV anatomy might be a subgroup of TAVR patients that could benefit from the use of such devices in cases of severe calcification (usually more represented when a raphe is present) or need for valve preparation and post-dilation.

This is the first meta-analysis comparing procedural complications within different morphologies with more than three studies for each outcome, and no difference was observed in the probability of the need for a second valve, surgery conversion, and aortic injury: within different BAV subtypes, TAVR seems to be confirmed as a feasible and safe approach. A higher risk of coronary compromise was observed by Du et al. in type 0 BAV, but their result was pooled from just two studies: we found that, even in large cohorts of patients, no event about coronary obstruction was reported in both groups, so we could not perform a diriment analysis. In the Yoon et al. study [29], excess leaflet calcification and moderate–severe calcification of raphes were independently associated with worse outcomes: if raphe calcification is obviously found in type 1 or 2 BAV, leaflet calcification could be also a type 0 property.

In the Yoon et al. study, excess leaflet calcification and moderate–severe calcification of raphes were independently associated with worse outcomes: if raphe calcification is obviously found in type 1 or 2 BAV, leaflet calcification could be also a type 0 property.

Pure valve morphology subtype by the Sievers classification seems to be poorly related to procedural outcomes; instead, CCT analysis of calcifications, raphes, and aortic morphology might be more reliable tools to predict complications and device success in the TAVR era for BAV, as stated in a previous study. There was not enough information about calcium location and severity in the analyzed studies to draw conclusions on procedural outcomes.

## 5. Limitations

It is essential to acknowledge certain limitations that may impact the interpretation of the findings. The included trials were of various designs and sizes, and all of them were retrospective. Inclusion and exclusion criteria were not uniform among the studies, along with the definitions of outcomes. Some of the studies did not differentiate between non-type 1 anatomies. Because of a lack of data about outcomes in subgroups like different prothesis types, severity, or distribution of calcium, it was not possible to perform further sub-analysis. Lastly, there was a paucity of data on long-term follow-ups, so we could not assess long-term differences.

## 6. Future Directions

Randomized controlled trials on long-term outcomes are warranted to promote widespread adoption of the percutaneous approach in BAV stenosis. However, nowadays, the first-line treatment for BAV patients remains surgical replacement, so a randomized comparison seems to be hard to achieve because physicians and patients might be unsure about participating. Nonetheless, the recent positive long-term results of trials comparing TAVR and SAVR in low-risk patients could be reassuring in proposing transcatheter replacement even in this population [70,71,72]. To achieve a truly useful insight into TAVR in BAV patients, even a retrospective analysis of different prosthetic platforms in different valve anatomies could be of important value. Also, investigating the long-term impact on hard endpoints of pacemaker implantation, paravalvular leakage, and post-procedural gradients might be of great interest to understand the targets for a truly efficient valve replacement.

## 7. Conclusions

TAVR in BAV patients seems to be an effective and safe alternative to SAVR concerning short-term outcomes except for the higher rates of permanent pacemaker implantation in the transcatheter intervention group. Bleeding events were found to be more frequent in patients undergoing SAVR. BAV anatomy can impact clinical outcomes in patients undergoing TAVR: in particular, Sievers type 1 morphology is associated with a higher probability of permanent pacemaker implantation but also with lower post-procedural transprothesis gradients.

## Figures and Tables

**Table 1:** Characteristics of included studies. Upper table: SAVR vs. TAVR studies; lower table: type 1 vs. non-type 1 studies. US = United States, PSM = propensity score match, NA = not available, UM = unmatched, TAVR = transcatheter aortic valve replacement, SAVR = surgical aortic valve replacement, N = number.

Study	Publication Year	Country	Adjustment	Patients (N)		
				Total	TAVR	SAVR
1-Gibson et al. [21]	2022	Ireland	NA	100	39	51
2-Majmundar et al. [22]	2021	US	PSM	2786	1393	1393
3-Sanaiha et al. [23]	2022	US	UM	56,331	3855	52,476
4-Soud et al. [24]	2020	US	PSM	136	68	68
5-Tsai et al. [25]	2020	Taiwan	Matched for age and sex	130	48	82
6-Elbadawi et al. [26]	2019	US	PSM	1950	975	975
7-Mentias et al. [27]	2020	US	PSM	1398	699	699
8-Husso et al. [28]	2021	Finland	PSM	150	75	75
**Study**	**Publication year**	**Country**	**Adjustment**	**Patients (N)**		
				Total	Type 1	Non Type 1
1-Yoon et al. [29]	2020	America	UM	1034	927	107
2-Jilaihawi et al. [30]	2016	Mixed	UM	95	74	21
3-Xiong et al. [31]	2018	Asia	UM	80	34	46
4-Forrest et al. [20]	2020	America	UM	150	136	14
5-Welixiang et al. [32]	2021	Asia	UM	181	79	102
6-Esposito et al. [33]	2022	Europe	UM	38	25	13
7-Jin et al. [34]	2022	Asia	UM	195	109	86
8-Ielasi et al. [35]	2020	Europe	UM	243	218	25
9-Fan et al. [36]	2020	Asia	UM	83	27	56

## Data Availability

No new data were created or analyzed in this study. Data sharing is not applicable to this article.

## Supplementary Materials

The following supporting information can be downloaded at https://www.mdpi.com/article/10.3390/jcm12237371/s1: Table S1: Baseline characteristics of patients, Table S2: Exclusion criteria of each study and definitions of the outcomes.

## List of Abbreviations

AKI: acute kidney injury; AS: aortic stenosis; BAS: bicuspid aortic stenosis, BAV: bicuspid aortic valve; CCT: cardiac computed tomography; CEPDs: cerebral embolic protection devices; LOS: length of stay; PPI: permanent pacemaker implantation; PVL: paravalvular leak; SAVR: surgical aortic valve replacement; TAV: tricuspid aortic stenosis; TAVI: transcatheter aortic valve implantation; TAVR: transcatheter aortic valve replacement.
