# Supplementary material for: A Meta-Analysis of Short-Term Outcomes of TAVR versus SAVR in Bicuspid Aortic Valve Stenosis and TAVR Results in Different Bicuspid Valve Anatomies
Source: J Clin Med. 2023 Nov 28;12(23):7371. doi: 10.3390/jcm12237371 (PMC10707011; doi:10.3390/jcm12237371)
Supplement: Supplementary file 1 [file jcm-12-07371-s001.zip › jcm-2741165-supplementary.pdf]

Supplementary Material

- **Table S1. Baseline characteristics of patients**
- **Table S2. Exclusion criteria of each study and Definitions of the outcomes**

Study	Age (Years) TAVR/SAVR	Female (%) TAVR/SAVR	STS-PROM (%)* TAVR/SAVR	EuroSCORE II(%)* TAVR/SAVR	Hypertension (%) TAVR/SAVR	Diabetes Mellitus (%) TAVR/SAVR	CKD (%) TAVR/SAVR	Heart Failure (%) TAVR/SAVR	CAD (%) TAVR/SAVR	PAD (%) TAVR/SAVR	Prior Stroke/TIA (%) TAVR/SAVR	Prior PCI (%) TAVR/SAVR	Prior CABG (%) TAVR/SAVR	Length of Stay (days)* TAVR/SAVR
Elbadawi et al[19]	65.7/65.2	40.0/36.4	-	-	64.6/64.6	29.7/30.8	19.5/24.1	4.6/6.2	10.3/8.7	27.7/30.3	9.2/11.3	8.7/7.2	10.3/8.7	4 (2-7)/7 (5-9)
Gibson et al[21]	71.0/53.0	46.0/34.0	-	2.93 ± 3/ 2.25 ± 3.6	49/33	31/6	-	-	38.0/8.0	3.0/6.0	8.0/6.0	-	-	3.5(±5) / 11 (±7)
Husso et al[28]	75.8/75.7	44/45.3	2.9 / 3.1	4.0 ± 3.3/ 3.8 ± 3.6	-	21.3/14.7	-	-	28.0/28.0	9.3/10.7	8.0/9.3	16.0/8.0	5.3/8.0	4.4 (±3.1)/8.0 (±6.0)
Majmundar et al[22]	68.3/68.1	37.8/38.4	-	-	78.8/75.6	28.0/29.4	23.8/23.8	67.1/66.0	10.2/9.7	26.4/25.5	6.8/6.8	10.2/9.7	5.8/6.7	2 (1-4) / 6 (5-8)
Mentias et al[27]	74.7/69.9	42.1/35	-	-	90.4/80.3	41.2/26.2	37/13.6	-	-	37.7/38	14.4/8.2	22.2/5.3	-	
Sanaiha et al[23]	69.0/59.0	48.9/40.8	-	-	-	26.7/15.6	-	67.1/26.7	45.9/22.7	20.1/32.8	-	-	4.8/0.4	2(1-6) / 6(4-8)
Tsai et al [25]	65.7/51.5	41.7/30.5	6.25 / 1.56	-	60.4/48.8	33.3/12.2	-	31.3/22.0	29.2/3.7	-	14.6/1.2	29.2/3.7	2.1/0	2.2 (±2.4) / 2.4 (±1.0)
Soud et al[24]	65.0/65.0	32.4/27.9			72.1/36.8	23.5/23.5	19.1/20.6	0.0/4.0	4.4/4.4	23.5/19.1	10.3/10.3			

Study	Age (Years) Type 1 / Type non 1	Female (%) Type 1 / Type non 1	STS-PROM Type 1 / Type non 1	Hypertension (%) Type 1 / Type non 1	Diabetes Mellitus (%) Type 1 / Type non 1	PAD (%) Type 1 / Type non 1	Prior Stroke/TIA (%) Type 1 / Type non 1	Prior PCI (%) Type 1 / Type non 1	BEV (%) Type 1 / Type non 1	Transprosthesic Gradient (mmHg)* Type1/Type non 1
Esposito et al[33]	-	-	-	-	-	-	-	-	-	
Fan et al[36]	78/75	41/42	4.7/5.1	48/53	10/14	-	4/0	18/5	11/0	
Forrest et al[18]	-	-	-	-	-	-	-	-	-	
Ielasi et al[35]	79/78	34/24	4.5/3.5	73/76	21/24	21/8	12/16	24/24	71/64	9.36(±4.7) / 11.53(± 6.7)
Jin et al[34]	76/73	59/53	4.5/4.1	53/37	16/20	15/7	7/7	7/7	0/0	12.25 (± 6.09)/ 12.76 ± (6.13)
Weixiang et al[32]	-	-	-	-	-	-	-	-	-	12.9 (± 7.0) / 16.3 (± 7.8)
Yoon et al [29]	75/70	59/59	3.8/3.0	76/80	28/24	12/14	11/13	19/19	70/83	10.4 (± 4.3) / 10.8 (± 5.4)
Jilaihawi et al[30]	76/74	38/48	4.9/4.2	-	20/38	-	12/14	11/19	-	10 (7.4-13) / 8 (7-13)
Xiong et al[31]	-	-	-	-	-	-	-	-	-	

Table S1. Baseline characteristics of patients. For references see the main manuscript.

STS PROM = Society of Thoracic Surgeons predicted risk of mortality, EuroSCORE = The European System for Cardiac Operative Risk Evaluation, TAVR = Transcathter aortic valve replacement, SAVR = Surgical aortic valve replacement, NA = Not available, BEV = Balloon Expandable Valve, CKD = chronic kidney disease, CAD = Coronary artery disease, PAD = Peripheral artery disease,TIA = Transient ischemic attack, PCI = Percutaneous coronary intervention, CABG = Coronary artery bypass grafting.

*Quantitative values are reported by mean (± standard deviation) or median (interquartile range).

Study	Pubblication year	Exclusion criteria
Elbadawi et al[26]	2019	age <18 years, concomitant aortic root repair, CABG, other valvulare surgeries, ventricular or atrial septal defect surgical closure, isolated aortic regurgitation, missing data for PSM variables or study outcomes
Gibson et al[21]	2022	age <18 years
Husso et al[28]	2021	age <18 years, previous surgical or transcatheter intervention on the aortic valve, acute endocarditis, isolated aortic valve regurgitation, or other major concomitant surgical procedures on other heart valves or thoracic aorta
Majmundar et al[22]	2021	age < 18 years, concomitant CABG, mitral, pulmonary, and tricuspid valve surgeries, atrial or ventricular sepal defect repair, and aortic root surgery, missing length of stay and those discharged before the necessary 30-day follow up period
Mentias et al[27]	2020	concomitant mitral valve surgery
Sanaiha et al[23]	2022	age <18 years, TAVR and SAVR during same hospitalization, aortic arch procedure, concomitant procedure, isolated aortic regurgitation, endocarditis, missing data, tricuspid geometry
Tsai et al [25]	2020	-
Soud et al[24]	2020	conccomitant procedures

Study	Pubblication year	Stroke	Acute kidney injury	Major bleeding	Permanent pacemaker placement
Elbadawi et al[26]	2019	ICD-9 or ICD-10	ICD-9 or ICD-10	ICD-9 or ICD-10	ICD-9 or ICD-10
Gibson et al[21]	2022	-	-	-	-
Husso et al[28]	2021	VARC II	KDIGO criteria, i.e., postoperative increase of creatinine ≥1.5 times, increase of creatinine ≥26.5 micromol/L or need for renal replacement therapy	E-CABG bleeding scores 2-3, i.e., transfusion of more than 4 units of red blood cells and/or reoperation for mediastinal and/or peripheral bleeding	-
Majmundar et al[22]	2021	ICD-10	ICD-10	a composite of gastrointestinal bleeding, post-operative bleeding, unspecified bleeding requiring blood transfusion	-
Mentias et al[27]	2020	-	-	-	-
Sanaiha et al[23]	2022	ICD-9 or ICD-10	ICD-9 or ICD-10	ICD-9 or ICD-10	ICD-9 or ICD-10
Tsai et al [25]	2020	-	-	-	-
Soud et al[24]	2020	VARC II	VARC II	VARC II	VARC II

Study	Pubblication year	Exclusion criteria
Jilaihawi et al[30]	2016	-
Xiong et al[31]	2018	permanent pacemaker at baseline
Yoon et al[29]	2020	tricuspid aortic stenosis, suboptimal CT quality, native pure aortic insufficiency
Fan et al[36]	2020	age <18 years, mortality before MRI, implantation of incompatible metallic prosthesis or foreign body contraindicated to the DW-MRI examination, including pacemaker implantation, history of a stroke or transient ischemic attack within the prior 6 months, absence of DW-MRI examinations for other reasons (i.e., in hospital death, conversion to SAVR, and unexplained CABG before the DW-MRI examination), intolerance due to clinical situation, and refusal of the DW-MRI examination or overscheduling, poor quality of imaging or out of the window period
Ielasi et al[35]	2020	type 2 BAV (according to Sievers classification), BAV type undeterminable
Weixiang et al[32]	2021	prior permanent pacemaker implantation, or ICD, without pre- and post-procedural enhanced CT or CT in poor quality, without sufficient electrocardiographic data to establish a 30-day high-degree atrioventricular block diagnosis
Forrest et al[18]	2021	age <60 years; any condition considered a contraindication for placement of a bioprosthetic valve (eg, subject is indicated for mechanical prosthetic valve); a known hypesensitivity or contraindication to any of the following that cannot be adequately premedicated: aspirin or heparin (HIT/HITTS) and bivalirudin, ticlopidine and clopidogrel, nitinol (titanium or nickel), contrast media; blood dyscrasias as defined: leukopenia (WBC <1000 cells/mm^3), thrombocytopenia (platelet count <50,000 cells/mm^3), history of bleeding diathesis or coagulopathy, or hypercoagulable states; ongoing sepsis, including active endocarditis; any percutaneous coronary or peripheral interventional procedure with a bare metal stent or drug eluting stent performed within 30 days prior to screening committee approval; multivessel coronary disease with a SYNTAX score >22 and/or unprotected left main coronary artery; symptomatic carotid or vertebral artery disease or successful treatment of carotid stenosis within 10 weeks of Heart Team assessment; cardiogenic shock manifested by low cardiac output, vasopressor dependence, or mechanical hemodynamic support; recent (within 2 months of Heart Team assessment) CVA or TIA; GI bleeding that would preclude anticoagulation; subject refuses a blood transfusion; severe dementia (resulting in either inability to provide informed consent for the study/procedure, prevents independent lifestyle outside of a chronic care facility, or will fundamentally complicate rehabilitation from the procedure or compliance with follow-up visits); estimated life expectancy of less than 24 months due to associated non-cardiac co-morbid conditions; other medical, social, or psychological conditions that in the opinion of the investigator precludes the subject from appropriate consent or adherence to the protocol required follow-up exams; currently participating in an investigational drug or another device study (excluding registries); evidence of an acute myocardial infarction ≤30 days before the study procedure due to unstable coronary artery disease (WHO criteria); need for emergency surgery for any reason; subject is pregnant or breast feeding; subject is legally incompetent, or otherwise vulnerable; anatomical exclusion criteria: pre-existing prosthetic heart valve in any position, severe mitral regurgitation amenable to surgical replacement or repair, severe tricuspid regurgitation amenable to surgical replacement or repair, moderate or severe mitral stenosis amenable to surgical replacement or repair, hypertrophic obstructive cardiomyopathy with left ventricular outflow gradient, prohibitive left ventricular outflow tract calcification, sinus of Valsalva diameter unsuitable for placement of the self-expanding bioprosthesis, aortic annulus diameter of <18 or >30 mm, significant ascending aortopathy requiring surgical repair, ascending aorta diameter >4.5 cm. For transfemoral or transaxillary (subclavian) access: access vessel mean diameter <5.0 mm for Evolut 23R, 26R, or 29R mm TAV, or access vessel mean diameter <5.5 mm for Evolut 34R mm or Evolut PRO 23R, 26R, 29 R mm TAV. However, for transaxillary (subclavian) access in patients with a patent LIMA, access vessel mean diameter <5.5mm for Evolut 23R, 26R, or 29R mm TAV, or access vessel mean diameter <6.0 mm for the Evolut 34R or Evolut PRO TAV
Jin et al [34]	2022	balloon-expandable valve, prosthetic valve

Esposito et al[33]	2022	tricuspid aortic stenosis

Study	Pubblication year	Stroke	Acute kidney injury	Major bleeding	Permanent pacemaker placement
Jilaihawi et al[30]	2016	VARC II	KDIGO (≥ stage 3)	-	VARC II
Xiong et al[31]	2018	-	-	-	VARC II
Yoon et al[29]	2020	VARC II	KDIGO (stage 2 and 3)	VARC II	VARC II
Fan et al[36]	2020	VARC II	-	-	VARC II
Ielasi et al[35]	2020	VARC II	-	VARC II	VARC II
Weixiang et al[32]	2021	-	-	-	Own
Forrest et al[18]	2021	mRS	KDIGO	BARC (>type 3b)	-
Jin et al [34]	2022	VARC III	VARC III	VARC III	VARC III
Esposito et al[33]	2022	-	-	-	ESC*

Table S2. Exclusion criteria of each study and Definitions of the outcomes. For references see the main manuscript.

CABG = Coronary artery bypass grafting, PSM = Proprensity match score, SAVR = Surgical aortic valve replacement, TAVR = Transcatheter aortic valve replacement, BAV = Bicuspid Aortic Valve, CABG = Coronary artery bypass grafting, CT = Computed Tomography, CVA = Cerebrovascular Accident DW-MRI = Diffusion Weighted Magnetic Resonance Imaging, GI = Gastrointestinal, HIT = Heparin Induced Thrombocytopenia, HITTS = Heparin Induced Thrombocytopenia and Thrombotic Syndrome, ICD = Implantable Cardioverter Defibrillator, LIMA = Left Internal Mammary Artery, MRI = Magnetic Resonance Imaging, PSM = Propensity match score, TAV= Tricuspid Aortic Valve, TIA = Transient Ischemic Attack, WBC = White Blood Count, WHO = World Health Organization, BARC = Bleeding Academic Research Consortiun, ESC = European Society of Cardiology, KDIGO = Kidney Disease Improving Global Outcomes, mRS = modified Ranking Scale, VARC = Valve Academic Research Consortium

*Conduction abnormalities as stated on ESC guidelines: new high degree AV block (complete or second-degree heart block), new onset LBBB with a a QRS duration >150 ms or PR >240 ms, pre-existing RBBB with new PR prolongation or change in axis
